# Oxidation Behavior of Tyranno ZMI-SiC Fiber/SiC-SiBC Matrix Composite from 800 to 1200 °C

**DOI:** 10.3390/ma11081367

**Published:** 2018-08-07

**Authors:** Donglin Zhao, Xiaomeng Fan, Xiaowei Yin, Xiaoyu Cao, Jing Zhang

**Affiliations:** Science and Technology on Thermostructural Composite Materials Laboratory, Northwestern Polytechnical University, Xi’an 710072, Shaanxi, China; zhaodonglin0@nwpu.edu.cn (D.Z.); yinxw@nwpu.edu.cn (X.Y.); caoxiaoyu@sust.edu.cn (X.C.); zj52sm@suho.com (J.Z.)

**Keywords:** ceramic matrix composite, fibers, oxidation resistance

## Abstract

In this work, the self-healing Si-B-C matrix-modified ZMI-SiC fiber/SiC (SiC/SiC-SiBC) composite was prepared by a combined process of chemical vapor infiltration, slurry infiltration, and liquid silicon infiltration. The weight changes of the as-fabricate composite after oxidation at 800, 1000, and 1200 °C for 100 h were −0.41%, −0.06%, +0.05%, respectively. The corresponding strength retention ratios were 75%, 99%, 100%, respectively. With the increase of oxidation temperature, coating cracks would be closed. A dense oxidation film was formed with the oxidation of the SiBC matrix, preventing the oxidation of interphase and fiber and leading to excellent oxidation resistance at 1000 and 1200 °C.

## 1. Introduction

Ceramic matrix composites (CMCs) have low density, high thermal shock resistance, and good oxidation resistance, so they show great potential as thermostructural materials [1,2,3,4]. For the application in aircraft engines, engine efficiency can be improved substantially due to the high operating temperature of CMCs [5]. The oxidation resistance of CMCs is important to long-term applications, like thermostructural parts in aircraft engines.

SiC fiber-reinforced silicon carbide matrix composite (SiC/SiC) shows excellent performance at high temperatures, so SiC/SiC is particularly attractive for high-performance aviation engine applications [6,7,8,9,10]. Chemical vapor infiltration (CVI) was usually employed to prepare SiC/SiC, but cracks and pores always exist in the matrix. The cracks resulted from the coefficient of thermal expansion (CTE) mismatch between fiber and matrix, and the pores resulted from the bottleneck effect of CVI. In the oxidation process, cracks and pores can be the inward diffusion channels of oxygen. In order to improve the oxidation resistance of SiC/SiC, cracks and pores should be filled to prevent the oxygen diffusion. Various kinds of self-healing phases were used to modify the interphase, matrix, and coating. The glassy phase, filling the cracks, can be formed at high temperatures with the oxidation of self-healing phases to inhibit the oxygen diffusion [11,12,13,14]. Up to now, the research focused on the preparation of self-healing matrix-modified SiC/SiC by CVI, and few reports can be found to introduce self-healing matrix into SiC/SiC by liquid silicon infiltration (LSI).

In the last decade, a combined process of CVI, slurry infiltration, and LSI has been developed to fabricate dense C/SiC containing SiBC matrix [15,16,17]. This combined process has two advantages, the decrease of porosity and the introduction of the self-healing matrix. Accordingly, it is expected to fabricate dense SiC/SiC incorporating SiBC matrix by the same process. In this paper, dense SiC/SiC-SiBC was prepared by the combined process, and then the oxidation behavior of SiC/SiC-SiBC at 800, 1000, and 1200 °C was studied. The oxidation behavior of SiC/SiC was also studied as a comparison.

## 2. Materials and Methods

### 2.1. Materials Preparation

**Figure 1:**
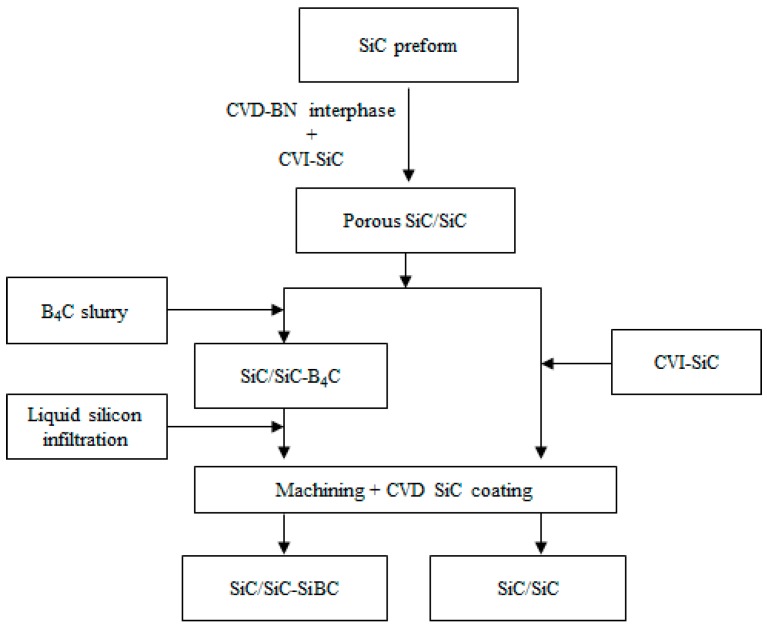
Schematic for the fabrication of SiC fiber-reinforced silicon carbide matrix composite (SiC/SiC) and self-healing Si-B-C matrix modified ZMI-SiC fiber/SiC (SiC/SiC-SiBC).

Tyranno ZMI-SiC fibers (56Si-1Zr-34C-9O, Ube Industries Ltd., Ube, Japan) were employed. The plain weave SiC cloths were stacked and fixed by graphite molds to prepare the SiC fiber preforms with a fiber fraction of 40 vol.%. The schematic for the preparation of SiC/SiC-SiBC and SiC/SiC is shown in Figure 1.

Firstly, the hexagonal boron nitride (h-BN) interphase was deposited on SiC fiber by chemical vapor deposition (CVD). The NH_3_-BCl_3_-H_2_-Ar was chosen as a gas source, and the deposition was carried out at 650 °С with a pressure of 1 kPa. After deposition of 80 h, the thickness of h-BN interphase was about 200 nm. For CMCs, the h-BN is an ideal choice for interphase, which can ensure crack deflection, and it had better oxidation resistance than pyrolytic carbon (PyC), so h-BN was chosen as interphase in this work. Secondly, the SiC matrix was introduced into the preform by CVI using methyltrichlorosilane (MTS) as a precursor, H_2,_ as a carrier gas, and argon as a dilution gas. The ratio of MTS/H_2_ was 1/10. The infiltration was carried out at 1000 °С with a pressure of 5 kPa. After infiltration of 240 h, the density of porous SiC/SiC reached to be 2.07 g/cm^3^. After that, the B_4_C particles were introduced into the specimens by slurry infiltration. The B_4_C powders (D_50_ = 1.5 µm, 50 wt.%) were dispersed into deionized water and then were ball-milled for 24 h to obtain the stable B_4_C slurry. The slurry infiltration was carried out under vacuum for 30 min. After drying at 120 °C, the excess slurry was removed from the surface. After repeated infiltration for four cycles, enough B_4_C particles were introduced into porous SiC/SiC, obtaining SiC/SiC-B_4_C. Thirdly, the specimens warped by silicon particles (Jinan Yinfeng Silicon Products Co., Ltd., Jinan, China) with an average size of 45 µm were placed in a graphite crucible. The crucible was put in a self-assembly vacuum furnace and heated rapidly to 1600 °C with a heating rate of 40 °C/min. The silicon powder melted at 1600 °C and spontaneously infiltrated into the preform by capillary force due to the good wettability between B_4_C particles and silicon. After holding at 1600 °C for 10 min, the furnace cooled down to room temperature. The residual silicon on the surface was removed, and the samples with desired dimensions were cut from the specimens. Finally, an SiC coating was prepared on the surface to protect the naked fibers and interphase, obtaining SiC/SiC-SiBC.

As a comparison, a CVI-based SiC matrix was continuously infiltrated into the porous SiC/SiC. After infiltration of 360 h, the samples with desired dimensions were cut from the specimens. At last, an SiC coating was prepared, obtaining SiC/SiC.

### 2.2. Characterization

According to the ASTM C-20 standard, the Archimedes method was employed to measure the open porosity and bulk density of specimens. The air oxidation tests were conducted for 100 h by muffle furnace at 800, 1000, and 1200 °C. The weight of samples before and after oxidation was measured by electronic balance (AG 204, Mettler Toledo, Greifense, Switzerland), with a range of 220 g and accuracy of 0.0001 g, and the weight was recorded after oxidation of 2, 10, 30, 50, 70, and 100 h during the tests. In each case, at least five samples were measured.

The samples with dimensions of 40 × 5 × 3 mm^3^ were used to measure the three-point flexural strengths by an universal testing machine (SANS CMT4304, Changchun, China) at room temperature; the span and loading rate are 30 mm and 0.5 mm/min.

The cross-section and fracture surface of samples were observed by a scanning electron microscope (SEM, S-2700, Hitachi, Tokyo, Japan) with the acceleration voltage of 15 kV and the working distance of 12.5 mm. Both secondary electron and backscattered electron were used to carry out the microstructure analysis. Prior to SEM analysis of cross-sections, the samples were carefully grinded with abrasive paper and polished with abrasive paste. An energy dispersive spectrometer (EDS, Genesis XM 2000, EDAX Inc., Mahwah, NJ, USA) was equipped with SEM to analyze the phase composition. The scanning time was 100 s to ensure the detection accuracy of EDS. X-ray diffraction (XRD, D/max-2400, Rigaku Corporation, Tokyo, Japan) with CuKα radiation was employed to analyze the phase composition of as-fabricated samples. The voltage was 40 kV and the current was 100 mA.

## 3. Results

### 3.1. Microstructure Analysis

The density and open porosity of SiC/SiC were 2.50 g/cm^3^ and 13 vol.%, and those of SiC/SiC-SiBC were 2.76 g/cm^3^ and 3 vol.%. Due to the introduction of a large amount of liquid silicon, SiC/SiC-SiBC showed lower open porosity than SiC/SiC, resulting in a higher density.

**Figure 2:**
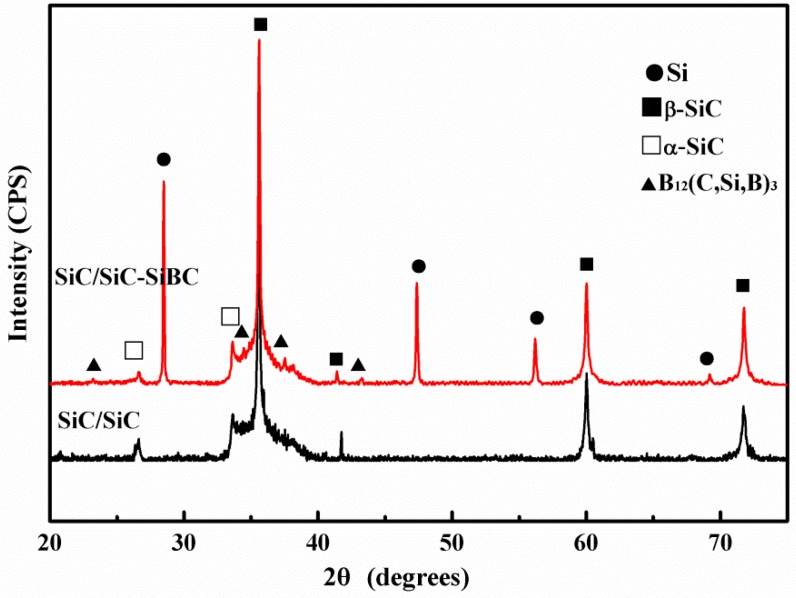
X-ray diffraction (XRD) patterns of SiC/SiC and SiC/SiC-SiBC.

**Figure 3:**
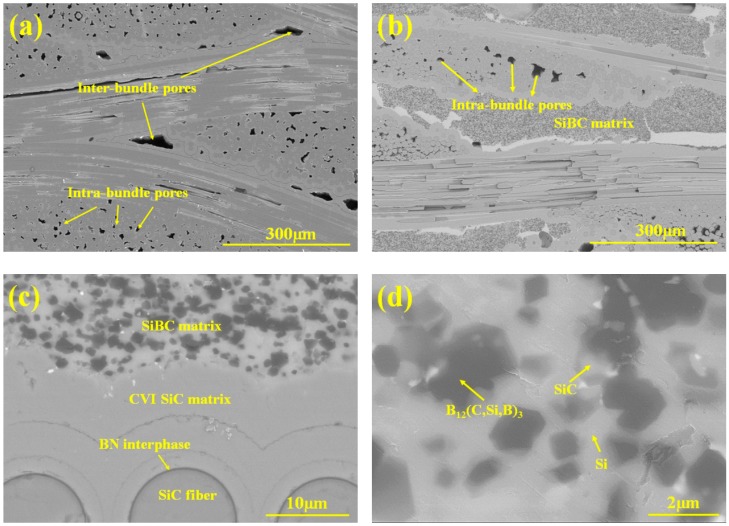

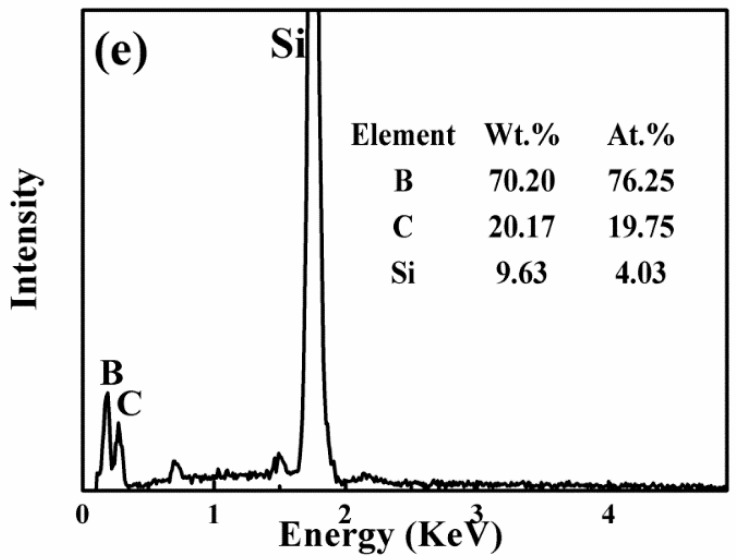
Low-magnification image of polished morphology of (**a**) SiC/SiC and (**b**) SiC/SiC-SiBC; High-magnification scanning electron microscope (SEM) images of (**c**) SiC/SiC-SiBC and (**d**) dense Si-B-C matrix; (**e**) the energy dispersive spectrometer (EDS) results of B_12_(C, Si, B)_3_ phase.

The XRD patterns of SiBC-modified SiC/SiC and SiC/SiC are shown in Figure 2. For SiC/SiC, there are only two kinds of SiC phase, α-SiC and β-SiC. While B_12_(C, Si, B)_3_ and residual Si can be found for SiC/SiC-SiBC besides α-SiC and β-SiC, due to the protection effect of SiC matrix, both composites have the same intrabundle microstructure, while the interbundle microstructure is different. The large interbundle pores remained in SiC/SiC (Figure 3a), while the SiBC-modified SiC/SiC had a dense interbundle matrix (Figure 3b). In the LSI process, due to the wettability between B_4_C particles and silicon, the silicon can spontaneously infiltrate the preform, and then dense SiBC matrix was formed by the reaction of B_4_C particles with silicon. As shown in Figure 3c, the SiC matrix protected the inner BN interphase and SiC fiber in the LSI process well. As shown in Figure 3d, the silicon, SiC, and B_12_(C, Si, B)_3_ formed the hybrid matrix and distributed homogenously in the dense SiBC matrix. The EDS results (Figure 3e) further confirmed the formation of the B_12_(C, Si, B)_3_ phase, which is consistent with the previous Reference [18]. Hayun et al. studied the reaction of B_4_C particles with liquid silicon, and found that the as-obtained microstructure was controlled by the dissolution–precipitation process [18]. For the infiltration of liquid silicon into the B_4_C preform, a core-rim structure was formed for B_4_C with a particle size of 100 µm, and a large amount of B_12_(C, Si, B)_3_ was formed for B_4_C with a particle size of 5 µm. In this work, the employed B_4_C has a particle size of 1.5 µm, so it can be concluded that the B_12_(C, Si, B)_3_ was formed by the reaction of B_4_C particles with liquid silicon.

### 3.2. Weight Variation

**Figure 4:**
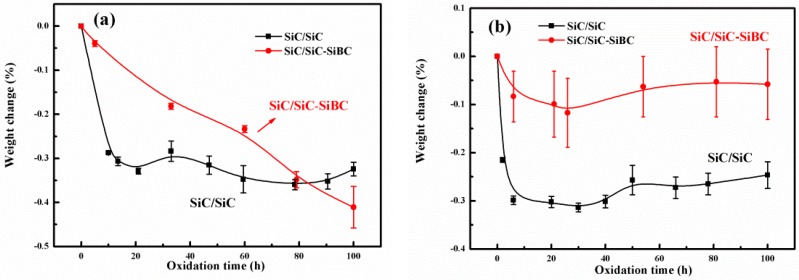

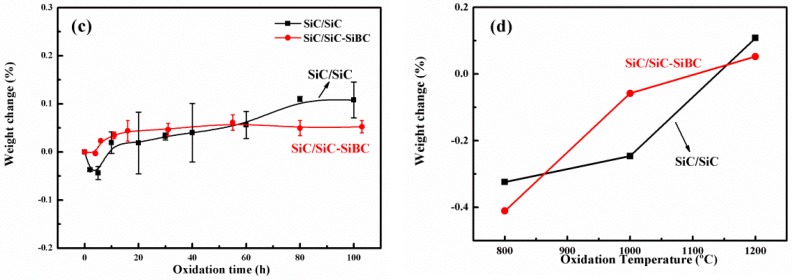
Weight variation of SiC/SiC and SiC/SiC-SiBC oxidized for 100 h at (**a**) 800 °C, (**b**) 1000 °C, (**c**) 1200 °C; (**d**) and the total weight variation for both two composites after oxidation of 100 h at different temperatures.

Figure 4 shows the weight changes of both two composites oxidized at different temperatures.

As shown in Figure 4a, as the oxidation time increased, the weight of SiC/SiC-SiBC decreased linearly at 800 °C, and weight loss reached to be 0.41% after the oxidation of 100 h. The weight of SiC/SiC decreased obviously at the initial 10 h, and then decreased slightly with the increase of oxidation time. As shown in Figure 4b, the weight of both composites decreased obviously at the initial 10 h for both composites oxidized at 1000 °C, and then decreased in a small range, with the oxidation time increasing. For the oxidation at 1200 °C (Figure 4c), both composites showed a slight decrease at the initial stage and then increased with the increase of oxidation time.

After oxidation of 100 h at 800, 1000, and 1200 °C, the weight changes of SiC/SiC were −0.32%, −0.24%, and +0.1%, while those of SiC/SiC-SiBC were −0.41%, −0.06%, and +0.05%, as shown in Figure 4d. With the oxidation temperature increasing, both composites showed the same transition from weight loss to weight gain, which can be attributed to the closure of cracks at 1000 °C.

Equations (1)–(8) list the possible reactions in the oxidation process.

SiC (s) + 3/2O_2_ (g) → SiO_2_ (s) + CO (g)     Δm = 50% (1)

Si(s) + O_2_(g) → SiO_2_ (s)     Δm = 114% (2)

2B_12_(C,Si,B)_3_ + 69/2O_2_ (g) → 15B_2_O_3_ (l) + 6SiO_2_ (l) + 6CO_2_ (g)     Δm = 165% (3)

2BN (s) + 3/2O_2_ (g) → B_2_O_3_ (l) + N_2_(g)     Δm = 42% (4)

B_2_O_3_ (l) + SiO_2_ (l) → B_2_O_3_·xSiO_2_ (l)     Δm = 0% (5)

B_2_O_3_·xSiO_2_ (l) → B_2_O_3_ (g)+SiO_2_ (l)     Δm = −53% (6)

C (s) + O_2_ (g) → CO_2_ (g)     Δm = −100% (7)

2C (s) + O_2_ (g) → 2CO (g)     Δm = −100% (8)

For SiC fiber, there was a 30 nm thickness carbon layer forming on the fiber surface after the desizing process [19]. As shown in the previous Reference [20], ZMI fiber is composed of nano-SiC grain, amorphous Si-C-O, and free carbon. So the weight loss by the oxidation of carbon can be obtained as shown in Equation (8).

Due to the low oxidation productivity of the BN interface, SiC matrix, SiBC matrix, and SiC coating at 800 °C, crack healing efficiency was very low. The oxygen can diffuse into the composites by the cracks, which led to the rapid oxidation of free carbon in SiC fiber, so both composites showed weight loss. At 1000 °C, the SiO_2_ and boron silicate glassy phases were generated by the oxidation of the SiBC matrix. The defects and cracks were healed by the glassy phases, which prevented the diffusion of oxygen, so the weight loss for the oxidation of 100 h was reduced. In previous work [17], the B_2_O_3_·*x*SiO_2_ glass was formed for the oxidation of SiBC matrix-modified C/SiC at 800 °C, while it was hard to detect the formation of SiO_2_ for the oxidation of SiC at 800 °C, revealing that the dense SiBC matrix has a quicker oxidation rate than the SiC matrix. So it is reasonable that the SiBC matrix-modified SiC/SiC shows smaller weight loss than SiC/SiC after the oxidation of 100 h at 1000 °C.

At 1200 °C, the self-healing phases were generated by the reaction of oxygen with the SiBC matrix. The defects and cracks were effectively healed by the self-healing phases, which prevented the inner diffusion of oxygen and protected the fiber and interphase from oxidation. It can be deduced that the weight gain rate by the oxidation of matrix and coating was greater than the weight loss rate by the fiber oxidation, so the weight of the SiBC matrix-modified SiC/SiC increased after oxidation of 100 h.

### 3.3. Residual Flexural Strength

**Figure 5:**
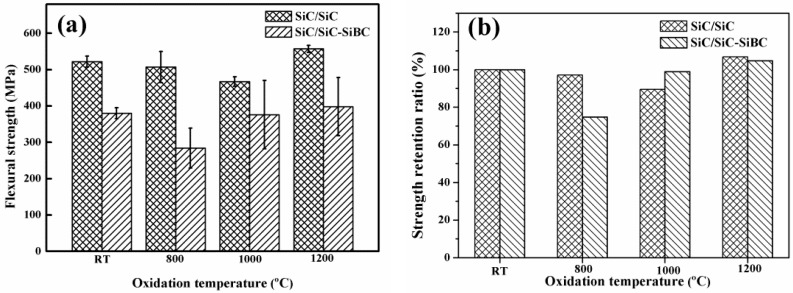
(**a**) The flexural strength and (**b**) the residual retention ratios of both composites after oxidation at different temperatures.

The room-temperature flexural strengths of the modified SiC/SiC and unmodified SiC/SiC were 380 ± 25 and 522 ± 15 MPa. The infiltration temperature of liquid silicon was 1600 °C, resulting in the damage of SiC fibers and the decrease of flexural strength. As shown in Figure 5a, the flexural strengths of SiC/SiC-SiBC oxidized at 800, 1000, and 1200 °C were 284 ± 55, 376 ± 45, and 398 ± 33 MPa, and those of SiC/SiC were 507 ± 43, 467 ± 13 and 557 ± 20 MPa. The corresponding strength retention ratios of SiC/SiC-SiBC were 75%, 99%, and 105% after oxidation, and those of SiC/SiC were 97%, 90%, and 107%, as shown in Figure 5b. All the strength retention ratios of SiC/SiC oxidized at different temperatures and were higher than 90%, revealing excellent oxidation resistance, while SiC/SiC-SiBC showed excellent oxidation resistance at 1000 and 1200 °C, except for oxidation at 800 °C.

There is the thermal residual stress (TRS) existing in composites, which would decrease the loading capacity of composites. In the oxidation process, the samples experienced several thermal shock cycles, which would relax TRS in the composites and improve flexural strength. So, the strength retention ratios higher than 100% appeared in this work.

**Figure 6:**
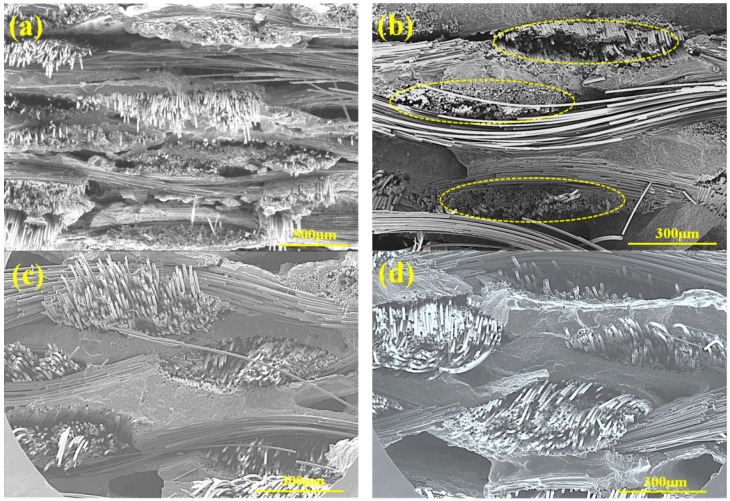
Fracture surface of (**a**) SiC/SiC after oxidation at 800 °C and SiC/SiC-SiBC after oxidation at (**b**) 800, (**c**) 1000, and (**d**) 1200 °C.

The fracture surface of both composites after oxidation at different temperatures was shown in Figure 6. For CMCs, the interphase is crucial to the loading capacity of fibers, so it can be deduced that good oxidation resistance means that the interphase and fibers were well protected in the oxidation process. As shown in Figure 6a, SiC/SiC showed the obvious fiber pullout, indicating that the h-BN interphase was protected well for the oxidation at 800 °C, which is consistent with high residual strength. A relatively flat fracture surface was obtained for SiC/SiC-SiBC after oxidation of 800 °C, and no obvious fiber pullout could be found, as marked in Figure 6b. At 800 °C, there would be liquid B_2_O_3_ and SiO_2_ formed by the oxidation of the h-BN interphase and SiC fiber, resulting in strong interphase bonding strength, so the SiC fiber cannot carry the load well, leading to low residual flexural strength. As shown in Figure 6c,d, the fiber pullout was clearly revealed in the fracture surface, indicating that the h-BN interphase and SiC fiber were protected well in the oxidation process. So the h-BN interphase can play a role in deflecting cracks, and the SiC fiber can carry the bearing load well, revealing high strength retention ratios after oxidation.

### 3.4. Oxidation Mechanism

**Figure 7:**
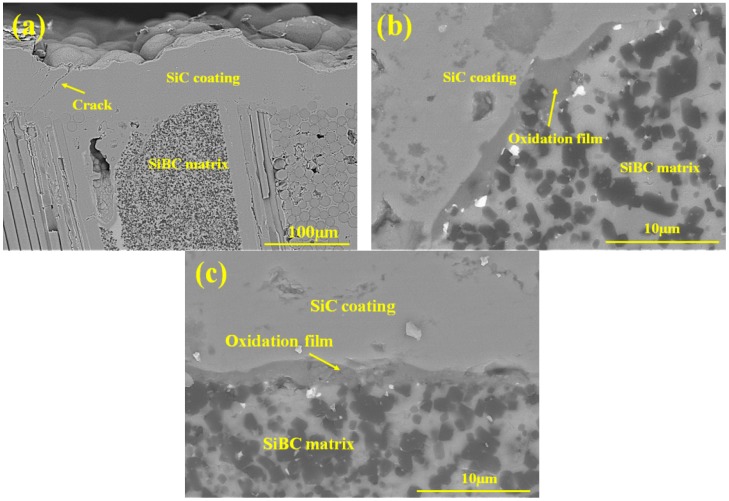
(**a**) Showing the coating crack of SiC/SiC-SiBC, (**b**,**c**) showing the dense oxidation film.

The cracks and pores in the composites are the diffusion channels for the oxygen. The matrix cracks and coating cracks were formed due to the CTEs mismatch between fiber, matrix, and coating. For both two composites, SiC coating was deposited at 1000 °C, so when the experienced temperature was higher than 1000 °C, coating cracks were closed spontaneously. Due to the CTE mismatch between SiC/SiC-SiBC and SiC coating, microcracks can be found in SiC coating, as shown in Figure 7a. Moreover, the SiBC matrix was oxidized to a form glassy phase to heal the cracks. For SiC/SiC-SiBC, SiC coating was deposited after machining, so part of BN interphase and SiC fiber contacted with the SiC coating directly. If there was any SiBC matrix beneath the coating, the SiBC matrix was oxidized to form the oxidation film (Figure 7b) to prevent the diffusion of the oxygen. Without an SiBC matrix beneath the coating, the oxygen would diffuse through the microcracks to erode the inner interphase and fibers, and then the interphase and fiber were gradually oxidized. So, the weight loss of SiBC matrix-modified SiC/SiC reached 0.41%, and the flexural strength decreased obviously after oxidation of 800 °C.

**Figure 8:**
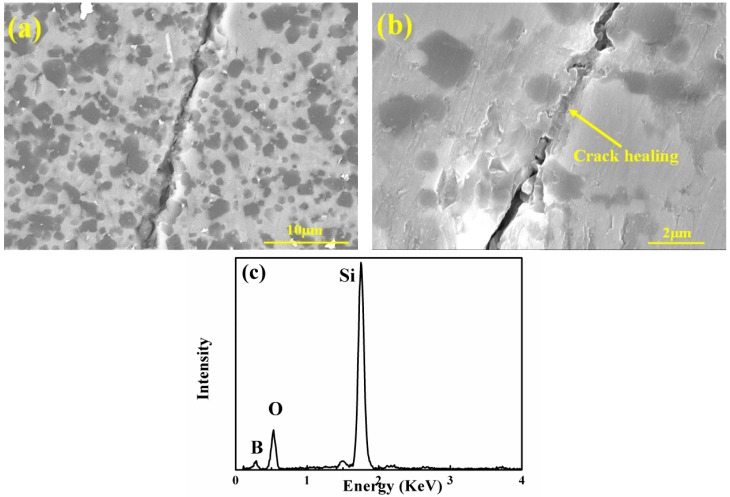
(**a**) Low-magnification and (**b**) high-magnification images showing the crack-healing phenomena, and (**c**) the EDS results of the glassy phase.

At 1000 and 1200 °C, the coating cracks would be closed, and the oxygen diffused through the coating defects and then reacted with SiBC matrix. The diffusion rate of oxygen to SiC coating was very low, and there would be enough time to form the oxidation film to protect the inner interphase and fiber (Figure 7c). At the same time, the matrix would play the self-healing effect to further inhibit the oxygen diffusion (Figure 8). So the inward diffusion of oxygen was hindered, and h-BN interphase and SiC fiber can be well protected, leading to slight weight variation of SiC/SiC-SiBC. At 1200 °C, the oxidation of the SiC coating led to slight weight gain after the oxidation of 100 h. Therefore, high strength retention ratios after the oxidation of 1000 and 1200 °C were contributed to the closing coating cracks and the self-healing matrix.

Compared with SiC/SiC, it can be found that oxidation resistance at 800 °C still needs to be improved, resulting from the CTEs mismatch between composites and coating. With the matrix densification and the introduction of self-healing phase, the CTE of as-fabricated composites changed, leading to CTEs mismatch, which is a problem for SiC/SiC containing SiBC matrix. In the future work, the oxidation behavior of SiC/SiC-SiBC will be optimized by tailoring the CTE match of fiber, matrix, and coating.

For SiC/SiC-SiBC, the thermal stability of Tyranno ZMI-SiC fiber also needs to be considered. In the present work, the lower flexural strength of the modified SiC/SiC than the unmodified SiC/SiC can be attributed to the damage of the ZMI fiber in the LSI process. In the future, it is expected to employ SiC fiber with better thermal stability to prepare SiC/SiC-SiBC with better mechanical properties and oxidation resistance. Moreover, the densification temperature may be reduced by the replacement of liquid silicon by Al-Si alloys to obtain high-performance SiC/SiC-SiBC [21].

## 4. Conclusions

SiBC matrix-modified SiC/SiC was prepared by a combined process of CVI, slurry infiltration, and LSI. After the oxidation of 100 h at 800 °C, the h-BN interphase and SiC fiber were not effectively protected, so a low-strength retention ratio of 75% was obtained. At high temperatures (1000~1200 °C), with the closure of coating cracks, and the formation of the glassy phase by the oxidation of SiBC matrix to fill the matrix cracks, the diffusion of oxygen was effectively hindered. The h-BN interphase and SiC fiber were well protected, so the strength retention ratios of 99% and 100% can be obtained after oxidation of 100 h at 1000 and 1200 °C.
